# Biobanking: Objectives, Requirements, and Future Challenges—Experiences from the Munich Vascular Biobank

**DOI:** 10.3390/jcm8020251

**Published:** 2019-02-16

**Authors:** Jaroslav Pelisek, Renate Hegenloh, Sabine Bauer, Susanne Metschl, Jessica Pauli, Nadiya Glukha, Albert Busch, Benedikt Reutersberg, Michael Kallmayer, Matthias Trenner, Heiko Wendorff, Pavlos Tsantilas, Sofie Schmid, Christoph Knappich, Christoph Schaeffer, Thomas Stadlbauer, Gabor Biro, Uta Wertern, Franz Meisner, Kerstin Stoklasa, Anna-Leonie Menges, Oksana Radu, Sabine Dallmann-Sieber, Angelos Karlas, Eva Knipfer, Christian Reeps, Alexander Zimmermann, Lars Maegdefessel, Hans-Henning Eckstein

**Affiliations:** 1 DZHK (German Centre for Cardiovascular Research), Munich Heart Alliance, 80636 Munich, Germany; Lars.Maegdefessel@tum.de (L.M.); HHEckstein@web.de (H.-H.E.); 2 Department of Vascular and Endovascular Surgery, Technische Universität München, 81675 Munich, Germany; renate.hegenloh@tum.de (R.H.); s.bauer@tum.de (S.B.); susanne.metschl@tum.de (S.M.); jessica.pauli@tum.de (J.P.); nadiya.glukha@tum.de (N.G.); Albert.Busch@mri.tum.de (A.B.); Benedikt.Reutersberg@mri.tum.de (B.R.); Michael.Kallmayer@mri.tum.de (M.K.); Matthias.Trenner@mri.tum.de (M.T.); Heiko.Wendorff@mri.tum.de (H.W.); Pavlos.Tsantilas@mri.tum.de (P.T.); sofie.schmid@mri.tum.de (S.S.) Christoph.Knappich@mri.tum.de (C.K.); Christoph.Schaeffer@mri.tum.de (C.S.) Thomas.Stadlbauer@mri.tum.de (T.S.); Gabor.Biro@mri.tum.de (G.B.); uta.werthern@mri.tum.de (U.W.); Franz.Meisner@mri.tum.de (F.M.); Kerstin.Stoklasa@mri.tum.de (K.S.); anna-leonie.menges@mri.tum.de (A.-L.M.); Oksana.Radu@mri.tum.de (O.R.); sabine.dallmann-sieber@mri.tum.de (S.D.-S.); Angelos.Karlas@mri.tum.de (A.K.); Eva.Knipfer@mri.tum.de (E.K.); Alexander.Zimmermann@mri.tum.de (A.Z.); 3 University Centre for Vascular Medicine and Department of Vascular Surgery, University Hospital Carl Gustav Carus, Dresden University of Technology, 01307 Dresden, Germany; Christian.Reeps@uniklinikum-dresden.de

**Keywords:** Munich Vascular Biobank, atherosclerosis, human vascular tissue, RIN, RNA fragmentation

## Abstract

Collecting biological tissue samples in a biobank grants a unique opportunity to validate diagnostic and therapeutic strategies for translational and clinical research. In the present work, we provide our long-standing experience in establishing and maintaining a biobank of vascular tissue samples, including the evaluation of tissue quality, especially in formalin-fixed paraffin-embedded specimens (FFPE). Our Munich Vascular Biobank includes, thus far, vascular biomaterial from patients with high-grade carotid artery stenosis (*n* = 1567), peripheral arterial disease (*n* = 703), and abdominal aortic aneurysm (*n* = 481) from our Department of Vascular and Endovascular Surgery (January 2004–December 2018). Vascular tissue samples are continuously processed and characterized to assess tissue morphology, histological quality, cellular composition, inflammation, calcification, neovascularization, and the content of elastin and collagen fibers. Atherosclerotic plaques are further classified in accordance with the American Heart Association (AHA), and plaque stability is determined. In order to assess the quality of RNA from FFPE tissue samples over time (2009–2018), RNA integrity number (RIN) and the extent of RNA fragmentation were evaluated. Expression analysis was performed with two housekeeping genes—glyceraldehyde 3-phosphate dehydrogenase (*GAPDH*) and beta-actin (*ACTB*)—using TaqMan-based quantitative reverse-transcription polymerase chain reaction (qRT)-PCR. FFPE biospecimens demonstrated unaltered RNA stability over time for up to 10 years. Furthermore, we provide a protocol for processing tissue samples in our Munich Vascular Biobank. In this work, we demonstrate that biobanking is an important tool not only for scientific research but also for clinical usage and personalized medicine.

## 1. Introduction

In general, biobanking is considered as a tool to collect and store biological tissue samples, mainly for research purposes. Management of a successful biobank requires a high level of collaboration and networking between various medical specialists, surgeons, biologists, and technicians to process the human specimens properly in time and to acquire not only biological but also clinical data. Biobanks are built to improve our understanding of various diseases and to help develop novel therapeutic strategies [1]. They are often the only sources of biological material available to explore uncommon human disorders, to solve the problems of heterogeneity of disease-related specific biomarkers, to determine the appropriate cut-off values, or to validate results achieved in animal experiments. Furthermore, new scientific approaches, such as next-generation sequencing or omics analyses, require high quality biospecimens and thus pose additional challenges for researchers [2,3,4].

In the last decades, new biobanks have been built focusing on selective human tissues, such as various cancer types or atherosclerotic specimens [1,2,3]. In this context, the collection of human atherosclerotic biomaterial following, for example, surgical intervention of stenotic carotid arteries or excision of aortic wall from patients with abdominal aortic aneurysm (AAA), has become a particularly important issue to improve our understanding of the underlying mechanisms leading to the formation and progression of atherosclerotic plaques and aneurysm toward vulnerable and rupture-prone phenotype. The first steps to collect vascular biospecimens from autopsies were performed in the 18th century by Rudolph Virchow, who explored inflammatory changes in atherosclerotic lesions [5]. It was not until a century later that another researcher, Russell Ross, summarized his histological observations on atherosclerotic tissue and postulated the famous response to injury theory of atherosclerosis [6]. Since then, other scientists have defined different stages of thrombus formation—occlusive thrombus, intraplaque thrombus, and transitional thrombus—and demonstrated plaque vulnerability to be a function of the reduced number of smooth muscle cells, increased inflammation, and size of the lipid core under a thin fibrous cap [7,8,9,10]. Renu Virmani et al. investigated coronary arteries from patients with sudden cardiac death and characterized additional plaque features, such as erosion, calcified nodules, fibrous cap atheroma, and fibrocalcific plaques [11]. All these studies were based on observations of large atherosclerotic tissue cohorts collected over years in the corresponding biobanks [12]. Thus, the collection of vascular tissue has clearly proven its importance and usefulness in discovering new features of atherosclerosis and factors contributing to plaque vulnerability [13,14,15].

The main challenge to run a biobank for accurate analyses, including next-generation sequencing and omics technologies, is to guarantee sufficient quality of the biospecimens. Universal methods for collecting, storing, and processing the obtained tissue samples are still lacking. These circumstances complicate translational insights as different biobanks are rarely directly comparable, and most studies focus on the results of their individual tissue and data collections. Thus, the number of specimen and patient data, especially long-term medical history, is always limited. In this work, we provide our long-standing experience in running a biobank of vascular tissue samples, including our analyses of tissue quality from FFPE samples, for successful and reliable expression analyses.

## 2. Experimental Section

### 2.1. Ethics Approval and Study Population

The permission to collect human carotid atherosclerotic, AAA, and peripheral aortic disease (PAD) biospecimens in our Munich Vascular Biobank was approved by the local Hospital Ethics Committee (2799/10, Ethikkommission der Fakultät für Medizin der Technischen Universität München, Munich, Germany). Written informed consent was obtained from all patients. Experiments were performed in accordance with the principles of the Declaration of Helsinki. The study population includes patients with high-grade carotid artery stenosis, PAD, and AAA, who underwent surgical intervention in our Department of Vascular and Endovascular Surgery in accordance with the current recommended guidelines [16,17,18]. 

### 2.2. Tissue Processing

A majority of the tissue samples were processed within two to four hours following surgical excision. According to our previous experiments and that of other researchers on RNA stability [19,20,21,22,23], no significant degradation of RNA is observed for up to eight hours at room temperature and 12–24 h on ice. In case of noncompliance, the time between excision and tissue processing was recorded, and these samples were used only for specific analyses where, for example, RNA stability is not the main issue.

Depending on size and quality of the tissue, consecutive segments were prepared, and the selected ones were directly frozen in liquid nitrogen and stored at −80 °C. The remaining segments were fixed in formalin overnight (minimum of 24 h) and decalcified using EDTA (0.2 M ethylenediaminetetraacetic acid in 50 mM tris(hydroxymethyl)aminomethane adjusted to pH 8.0) for a couple of days depending on the extent of calcification. Afterward, the tissue segments were embedded in paraffin and stored at room temperature. From all segments, consecutive sections of 2–3 µm were prepared and stained with haematoxylin–eosin (HE) and elastin–van Gieson (EvG) to assess the tissue morphology. 

The individual histological features were graduated semiquantitatively by independent assessors as follows: (-) no staining; (+) positive staining of most cells or the analyzed components of vessel wall in a majority of specimens; (++) medium overall positive staining detected in all cells/vessel wall components in all specimens; (+++) strong overall positive staining detected in all cells/vessel wall components in all specimens; (-/+) intensity of staining varied between different samples and also within the same specimen; (-/++) heterogeneous staining results from (-) to (++). 

### 2.3. RNA Extraction from FFPE Biospecimens by Expression of Housekeeping Genes

Total RNA was isolated from FFPE tissue sections (4 × 20 µm thickness) adjacent to the slides applied for histological characterizations using the High Pure RNA Paraffin Kit in accordance with the manufacturer’s instructions (Roche, Mannheim, Germany). The amount and the purity of RNA were determined using Nanodrop 2000 (Thermo Fisher Scientific, Munich, Germany). 

### 2.4. Analysis of RNA Quality from FFPE Biospecimens by RIN and RNA Fragmentation

**Figure 1:**
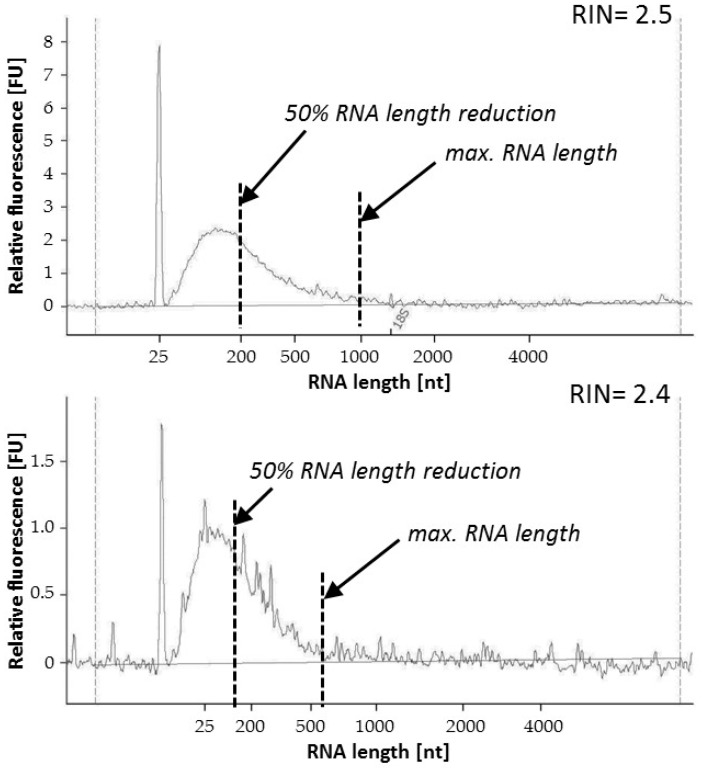
Evaluation of the extent of RNA fragmentation from FFPE tissue samples measuring the area under the curve from Agilent Bioanalyzer. Two values were defined: maximal RNA length and the 50% RNA length calculated as a 50% reduction of the area under the curve. RIN: RNA integrity number; FFPE: formalin-fixed paraffin-embedded specimens. nt: number of nucleotides; FU: fluorescence unit.

RNA integrity number (RIN) was determined by Agilent 2100 Bioanalyzer and the RNA 6000 Nano Kit (Agilent Technologies, Waldbronn, Germany) in accordance with the manufacturer’s instructions. In order to receive additional information about the quality of RNA, the length of the RNA fragments was evaluated from the data curve of the Bioanalyzer, calculating the maximal length and the RNA fragment size at 50% reduction of the area under the curve (Figure 1). 

### 2.5. Analysis of RNA Quality from FFPE Biospecimens by Expression of Home-Keeping Genes

For PCR analysis, RNA was reverse-transcribed into complementary DNA (cDNA) using random hexamer primers and cDNA Synthesis Kit RevertAid (Fermentas, St. Leon-Rot, Germany). Quantitative qRT-PCR was performed using StepOnePlus RT-PCR-System (Applied Biosystems/Life Technologies, Darmstadt, Germany) and TaqMan primers for two housekeeping genes—glyceraldehyde 3-phosphate dehydrogenase (*GAPDH*, Hs04420697_g1, 130 bp) and beta-actin (*ACTB*, Hs01060665_g1, 63 bp)—to determine the gene expression level from various tissues samples over time between 2009 and 2018. 

### 2.6. Statistical Analysis

For statistical analysis, SPSS for Windows version 20.0 (SPSS Inc., Chicago, IL, USA) was applied. Kruskal–Wallis test was used to set statistically significant differences between all study groups. The nonparametric Mann–Whitney *U* test was applied as a post-hoc comparison between the interindividual values. A *p*-value of <0.05 was considered as statistically significant.

## 3. Results

### 3.1. Study Population

**Figure 2:**
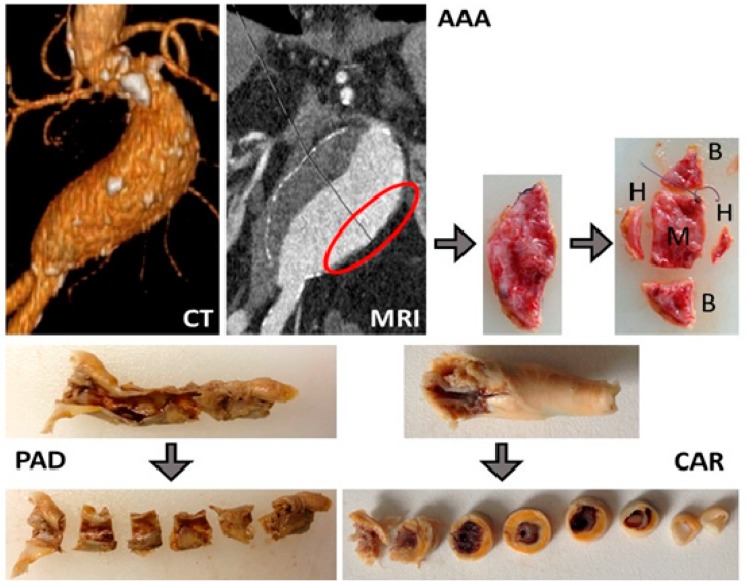
Examples of tissue samples collected in our Munich Vascular Biobank. CAR: carotid atherosclerotic plaques obtained from patients with high-grade carotid artery stenosis (>50%) [16] by endarterectomy (EA); PAD: atherosclerotic plaques from patients with peripheral artery disease obtained by EA [17]; AAA: aortic wall from patients with abdominal aortic aneurysm who underwent open surgical repair [18]; B: fresh frozen segments for molecular biology; H: histology; M: mechanics (tensile tests); CT: computer tomography, MRI: magnetic resonance imaging.

Starting in January 2004 until December 2018, vascular tissue was collected from over 2700 patients (carotid plaques, *n* = 1567; AAA wall specimens, n = 481; PAD, *n* = 703, since 2016, including peripheral aneurysms, *n* = 64 and thrombus, *n* = 80) (Figure 2, Table 1). Furthermore, serum was collected from over 4400 patients (patients with high-grade carotid stenosis, *n* = 1394; AAA patients, *n* = 1380; PAD patients, *n* = 1702). For comparison, we also collected healthy aortic tissue samples as control from the Department of Trauma Surgery following kidney transplantation (*n* = 102). In addition, 15 healthy carotid arteries were obtained from the Department of Forensic Medicine.

In addition, clinical data from all patients whose tissue was collected in our Munich Vascular Biobank were acquired (if available), including age, sex, medication, accompanying diseases, and findings from the Department of Clinical Chemistry. The collection of vascular tissue samples has proved its usefulness leading to plethora of scientific publications (Table 2) and helping to discover new features contributing to AAA, PAD and carotid plaque vulnerability.

### 3.2. Tissue Processing and Characterization

For a better understanding of the development of atherosclerotic plaques or AAA, and to link these findings with patient medical history and accompanying diseases, a large number of specimens is necessary. Such endeavor requires good organization and logistics and is time-consuming. A particularly critical issue here is contemporary, accurate, and fast tissue processing. Furthermore, additional work is necessary, such as continuous plaque characterization and acquirement of available patient data, especially regarding the correct diagnosis. 

**Figure 3:**
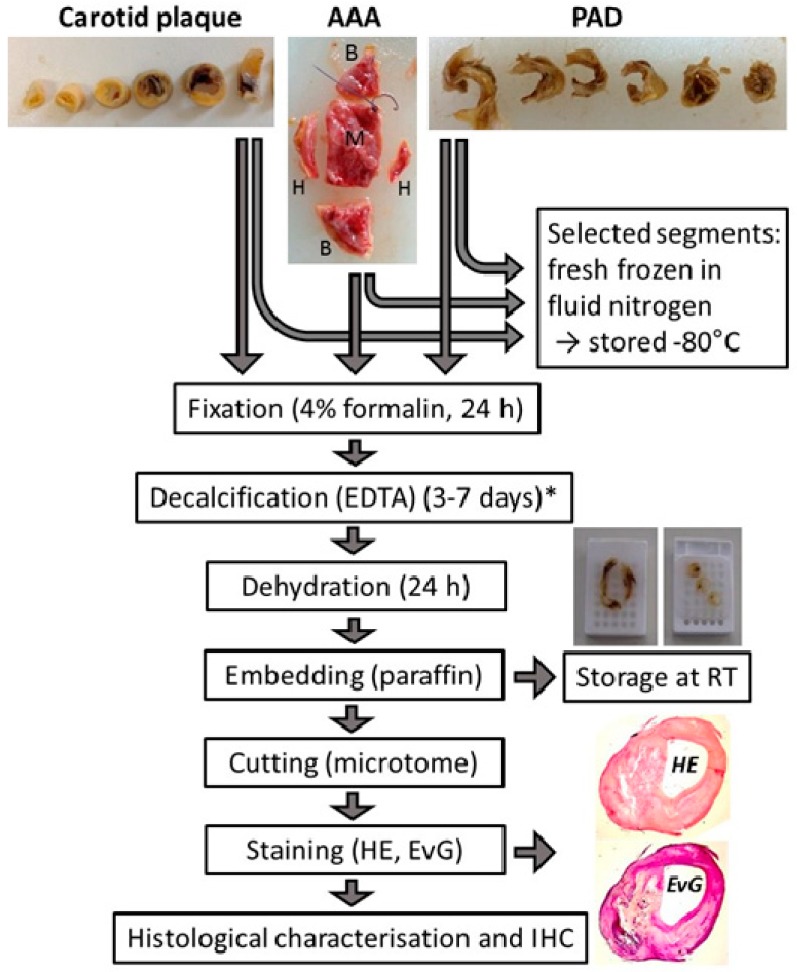
Schematic chart of the processing of vascular tissue after surgical excision. Carotid plaque: atherosclerotic lesions from patients with high-graded carotid artery stenosis; AAA: aortic wall from patients with abdominal aortic aneurysm; PAD: atherosclerotic tissue from patients with peripheral artery disease; RT: room temperature; IHC: immunohistochemistry. HE: haematoxilin-eosin staining; EvG: elastica van gieson staining; * the time depends on the extent of calcification.

Here, we provide an example of the tissue sample processing in our Munich Vascular Biobank (Figure 3):

Carotid artery tissue biospecimens: Following surgical excision, the complete atherosclerotic plaque is divided into segments of 3–4 mm each (mostly 3–7, depending on the size of the excised plaque tissue). Selected segments are immediately frozen and stored for molecular analyses. The remaining segments are fixed in formalin, decalcified, and embedded in paraffin as described in the Experimental Section. In this way, over 5000 carotid atherosclerotic plaque segments of different stages of atherosclerosis have been collected. From all segments, consecutive sections are continuously prepared, stained, and characterized to assess histological quality, cellular composition, the degree of infiltration with inflammatory cells, stage and extent of calcification, neovascularization, and the content of elastin and collagen fibers.

**Figure 4:**
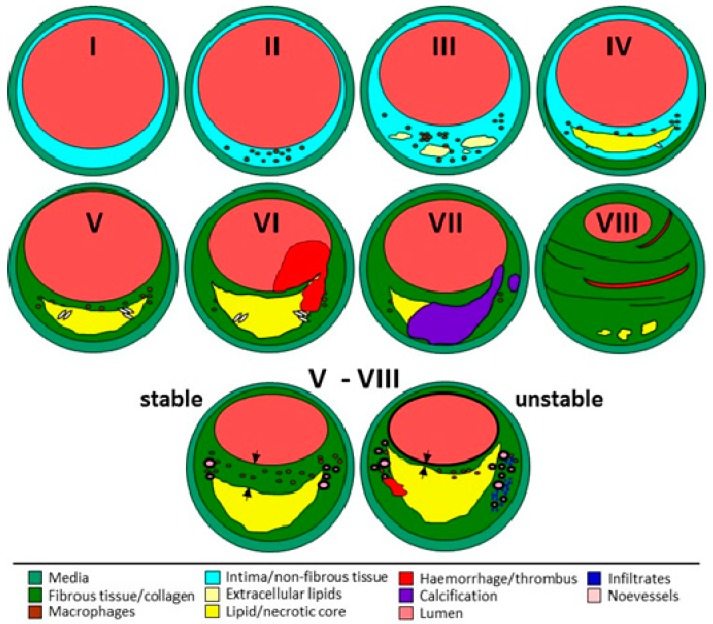
Classification of atherosclerotic lesions according to the American Heart Association (AHA) [24,25,26,27]. Type I: initial lesion with isolated macrophages and macrophage-derived foam cells; type II: fatty streaks, increased number of foam cells, intracellular lipid accumulation; type III: further accumulation of inflammatory cells and intracellular lipids, isolated extracellular lipid deposits; type IV: atheroma, formation of confluent lipid core without perceptible fibrous cap; type V: fibroatheroma, formation of fibrous layer over the lipid/necrotic core; type VI: as V but with thrombus and/or intraplaque hemorrhage; type VII: as V with calcified nodules, calcification predominates; type VIII: fibrous tissue predominates, lumen mainly small, lipid deposits minimal or absent. Plaque stability was assessed in line with [28]: thin fibrous cap <>200 µm (arrows) over a larger necrotic core. Unstable/vulnerable plaque can develop from each plaque type of type V–VIII. Modified from [29].

Furthermore, the atherosclerotic segments are classified in accordance with the AHA [24,25,26,27] to assess the type of atherosclerosis (Figure 4). In addition, plaque stability is determined in line with the criteria outlined by Redgrave and Rothwell [28], i.e., stable lesions are defined as plaques with a thick-cap fibroatheroma of >200 µm over a lipid/necrotic core (or without), while unstable lesions are defined either as ruptured or as plaques with thin-cap fibroatheroma of <200 µm over a large necrotic core.

Peripheral artery tissue biospecimens: These tissue samples resemble carotid plaques [30] and are treated in a similar way as described above. However, plaque stability is not assessed, and plaque classification is performed only for selected tissue samples [30]. Furthermore, a majority of these biospecimens are FFPE-treated with a focus on histology.

AAA tissue biospecimens: Following surgical excision of a piece of the diseased aortic wall from the left anterior section of the aneurysm, the tissue is divided for histological and molecular biological analyses, as shown in Figure 3. A selected number of AAA wall samples also undergo tensile tests (M) to obtain additional information about the mechanical properties of the AAA wall [31,32,33,34,35]. Appointed segments (Figure 3) are immediately frozen in liquid nitrogen and stored accordingly (B). The remaining segments (H) are fixed in formalin, decalcified, embedded in paraffin, and stored at room temperature as described in the Experimental Section. Again, consecutive slices of 2–3 µm are prepared from all FFPE segments, stained, and characterized to assess tissue morphology, quality, and other histological features as described above.

### 3.3. Analysis of RNA Quality from FFPE Biospecimens by RNA Integrity Number

**Figure 5:**
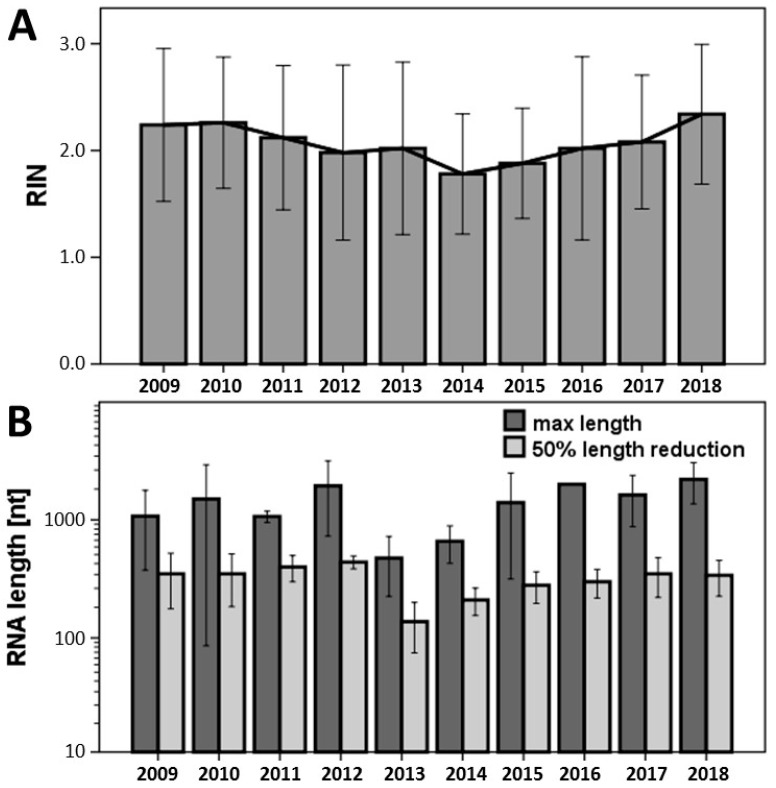
(**A**) Measurement of RNA integrity number (RIN) in FFPE vascular tissue samples using Agilent Bioanalyzer between 2009 and 2018 (*n* = 5 for each group and year). (**B**) Evaluation of the length of the RNA fragments, as described in Figure 3. No significant differences were observed between the study years over time.

Because most of our vascular tissue samples are FFPE, in this work, we focused on the suitability of these biospecimens for expression analysis. The RNA quality stated by the RIN was unchanged for all time points for up to 10 years back, without any significant differences between the individual tested years (Figure 5A). The average RIN from all FFPE biospecimens analyzed in the study was 2.0 ± 0.6.

### 3.4. Analysis of RNA Quality from FFPE Biospecimens Evaluating RNA Fragmentation

Independent of RIN, which is determined by comparing the degradation of 18S and 28S RNAs, the mRNA of interest can be differentially fragmented independently of the mentioned ribosomal RNAs. Thus, measuring the overall RNA fragmentation may provide additional information about the RNA quality and the maximal and average length (50% of the area under the curve from the Bioanalyzer, Figure 1) of RNA fragments in all tissue sample analyzed, as also partially described by Illumina [36]. The maximal length of the RNA of all vascular biospecimens exceeded 500 nt, while most of them were even longer than 1000 nt (Figure 5B). Calculating the drop-down of 50% showed that more than half of the RNA fragments in FFPE tissue samples was still longer than 200 nt. Furthermore, no significant differences in the overall RNA fragmentation were observed for up to 10 years back.

### 3.5. Analysis of RNA Quality from FFPE Biospecimens by Expression of Housekeeping Genes

**Figure 6:**
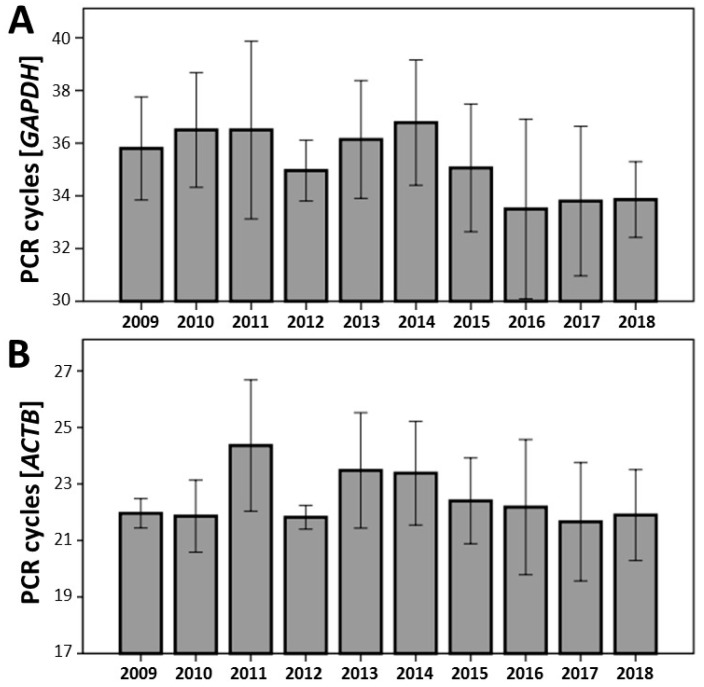
Results of qRT-PCR analysis from FFPE vascular tissue samples from different years between 2009 and 2018 (*n* = 5 for each study group) using TaqMan primer for glyceraldehyde 3-phosphate dehydrogenase (**A**, *GAPDH*, 130 bp) and beta-actin (**B**, *ACTB*, 63 bp). No significant differences were observed between the individual years over time.

Independent of RIN values or the degree of RNA degradation to assess the quality of RNA, expression analysis using various housekeeping genes is another important and helpful measure to prove the suitability of tissue samples for analyses, such as for expression analysis. Consequently, independent of the results from the Bioanalyzer (RIN, overall RNA fragmentation), we evaluated the expression of two housekeeping genes: *GAPDH* and *ACTB* (Figure 6). The mRNA expression from the selected FFPE vascular tissues was detected in all biospecimens. Using the same threshold for all PCR reactions, no significant differences were observed for cycles crossing the chosen threshold, independent of the selected year (up to 10 years back). The expression at mRNA level depended only on the concentration of the total amount of extracted RNA used in the study (data not shown). Furthermore, no significant relationship was observed between RIN, total RNA fragmentation, and mRNA expression.

## 4. Discussion

The high quality of biospecimens is an important factor for consistent and reliable analysis, and global harmonization of corresponding protocols is necessary to reduce variability between different biobanks [37,38,39]. The critical step is the time span between surgical excision and tissue preservation, which markedly influences, in particular, the quality of RNA in the acquired tissue samples [19,20,21,22,23]. In addition, to ensure the high quality of the biospecimens, suitable tests should be developed and applied to assess the quality of the biospecimens for the desired experiments. Unfortunately, a majority of publications dealing with tissue material from various biobanks do not cite how the biospecimens were obtained and how they were processed [40]. The most commonly used techniques for preservation of biomaterial are based on hypothermic or chemical form of treatment. Though both procedures are considered the most suitable methods for storage, they have their drawbacks, which might hamper the quality of the biospecimens and consequently the possible outcome of the results [41,42,43]. 

The RNA integrity is frequently used as a measure of tissue quality because RNA particularly rapidly degrades after tissue excision or after death due to its highly unstable nature. However, in contrast to the vulnerable purified RNA accessible to RNases, which is ubiquitously present, the RNA in tissue seems to be more stable [44]. A plethora of studies have already found that the RNA is stable within the tissue for several hours even at room temperature [22,44,45,46]. RNA was stable for up to three hours at RT in human breast biopsies [45], for up to five hours in human lung tissue [46], for up to 16 h on ice or at RT in human tonsil and colon tissue [44], and for up to 12 h at RT and 24 h on ice in liver tissue [22]. In addition, the RNA integrity is not significantly decreased after surgery [47]. Nevertheless, depending on the tissue samples stored in the individual biobanks and the time span between surgical excision and sample processing, the integrity of RNA should be tested beforehand to guarantee reliable results, even if such a verification is time-consuming and costly. 

Many biobanks around the world are driven by institutes of pathology that commonly obtain tissue samples from different surgical departments to issue diagnostic findings and proper therapeutic strategies. Fixation, particularly in formalin, followed by embedding in paraffin (FFPE) is a very common technique to preserve human tissue samples. Consequently, FFPE tissue samples represent a great challenge in molecular biology because the fixation process causes nucleic acid degradation, resulting in fragmented RNA transcripts [48,49]. A common method to assess the integrity of RNA used by many researchers is the measurement of the RIN [50]. Our results demonstrated no significant differences in RIN for up to 10 years back, indicating that the RNA is stable over time even if the FFPE biospecimens are stored at room temperature. In this work, we also proposed an additional method to evaluate the RNA quality by assessing the degree of total RNA fragmentation. The determination of the maximal length of all the RNA fragments and 50% drop-down confirmed that no significant degradation of RNA in FFPE tissue samples occurred over time. Many of the biospecimens had maximal available RNA length over 1000 nt. Taking into account the 50% reduction of the RNA length, a majority of the tissue samples still contained RNA that were more than 200 nt long. Thus, in accordance with Illumina RNA enrichment assays and recommendations [36], these samples might also be suitable for RNA sequencing.

In order to further support our assumption that FFPE tissue samples are suitable for expression analyses at the mRNA level, we tested two different housekeeping genes: *GAPDH* and *ACTB*. Again, independent of the RIN value (even if lower than 1) or extended overall RNA fragmentation, expression of these genes could be detected in all FFPE biospecimens. Furthermore, no significant differences in the expression were observed over time, confirming the tissue stability and the fact that the storage of FFPE tissue samples at RT was adequate. No significant correlation was found between RIN values, global RNA fragmentation, and gene expression. The expression level depended only on the amount of RNA extracted from each sample. These results accredit that FFPE samples can be used without any concerns for expression analyses as well. However, the PCR amplicons should be kept short (<150 nt, preferably <100 nt) [51]. Furthermore, RIN measurement and RNA fragmentation analysis from the Bioanalyzer traces might be helpful in evaluating the quality of RNA and to exclude potential samples unsuitable for the intended experiments. In addition, due to the RNA degradation in FFPE tissue, hexamer primers should be used to prepare cDNA for RT-qPCR [22]. Using oligo (dT) primer for reverse transcription might lead to inconsistent results because poly A tail might be missing in many mRNA molecules, which also depends on the length and stability of the targeted mRNA. It should also be mentioned that the degradation of mRNA from various genes might differ from the housekeeping genes used in our study. Furthermore, genes expressed at high level, such as the housekeeping genes, may not work optimally to measure subtle differences in quality [52,53]. Thus, testing of various primer pairs from different mRNA regions for each individual transcript of interest is recommended to achieve reliable results.

In order to link the results from the vascular tissue to the patient’s clinical data, histological features from all segments of each patient were compared to select the most clinically relevant characteristics. The following criteria and priorities were applied [29,54,55]: (i) plaque vulnerability (unstable > stable), (ii) plaque type (complex plaque > VI >V > VII), (iii) inflammation (positive > absent), and (iv) content of collagenous fibers (absent > positive). In this manner, we compared atherosclerotic plaques with sex, age, and history of neurological symptoms in 763 patients with high-grade carotid artery stenosis [54]. Male sex was significantly associated with increased inflammation and neovascularization. Higher age correlated with calcification, and unstable plaques were found more frequently in symptomatic patients. Interestingly, plaque morphology significantly differed between men and women and continuously changed with age, even though it was less striking than expected. Furthermore, our results showed that, independent of age, asymptomatic men had unstable plaques more frequently than women [55]. Thus, apart from age, male sex particularly seems to be an additional risk factor for ischemic stroke.

Based on our long-standing experience, each institute or research group intending to run a biobank should consider the following items before starting such a complex and ambitious endeavor: (i) A decision must be made on what tissue samples are to be collected and for what purpose. In this context, potential future perspectives and intentions should be considered, e.g., next-generation sequencing, omics analyses, etc. (ii) An organizational chart should be prepared in line with the corresponding surgical departments, medical specialists, pathologists, and/or biologists. (iii) The logistics is a critical item that should be considered to ensure the contemporary transfer of the biomaterial after tissue excision, its processing, and preservation. (iv) Standard protocols should be established for proper tissue processing, segmentation, and preservation techniques (cryopreservation, fixation with formalin, etc.). (v) Proper storage should be ensured for individual tissue samples at all times. FFPE can be stored at RT, as shown in our study. Fresh frozen tissue samples should be stored at a minimum of −80 °C; even better would be to store them at −150 °C (cryogenic freezer) or in liquid nitrogen tanks (−196 °C). (vi) Tissue collection alone does not make a good biobank; data acquisition from the patients, including medical history, accompanying diseases and other available datasets are important and helpful tools to link the results from tissue analyses to the corresponding diseases. (vii) Obtaining adequate control tissue samples is another critical point. Healthy individuals rarely undergo surgical intervention, so the access is very limited. (viii) Last but not least, ethical approval from the local ethics committee and permission from each patient to collect the tissue samples are necessary, along with the General Data Protection Regulation to guarantee patient privacy. All data should be appropriately protected, and only authorized persons should have access.

## 5. Conclusions

Biobanking is the most appropriate library of biological materials for not only scientific research but also for clinical usage. The accuracy of the data generated from the biobank strongly depends on the quality of the stored tissue samples. Thus, the major challenge is the standardization of protocols for biospecimen processing and preservation.

In this work, we summarized our own experiences in managing a vascular biobank, starting with tissue excision through different processing techniques, testing the quality of the biospecimens, up to their proper storage. Furthermore, we provided evidence that FFPE tissue samples are suitable not only for histological and immunohistochemical analyses but also for expression analyses at the mRNA level and potential RNA sequencing. In addition, apart from RIN, we offered an additional tool to analyze the quality of tissue samples by calculating the extent of the RNA fragmentation. Such an approach ensures accurate selection of suitable samples for the desired experimental design. 

Biobank of the future is facing many challenges to be able to discover, develop, and properly validate new diagnostic and therapeutic strategies for basic, translational, and clinical research. Biobanking of high-quality human biospecimens, together with patient clinical information, provides a fundamental scientific infrastructure for personalized medicine.

## Figures and Tables

**Table 1:** Munich Vascular Biobank. List of samples collected over the years.

Year	AAA ^#^		CAROTIS		PAD	
	FFPE Tissue	Serum	FFPE Tissue	Serum	FFPE Tissue	Serum
**2004**			79	47		
**2005**	8	17	72	63		
**2006**	36	84	36	84		
**2007**	40	89	79	56		
**2008**	34	84	63	72		
**2009**	40	60	100	62	63	90
**2010**	40	72	94	58	77	78
**2011**	38	91	121	87	51	133
**2012**	33	136	122	111	62	251
**2013**	41	114	126	99	63	228
**2014**	24	131	92	97	63	259
**2015**	26	126	126	131	84	294
**2016**	30	124	124	117	80/27/35 *	228
**2017**	48	127	173	156	22/15/23 **	116
**2018**	43	125	160	154	16/22 **	25
**Sum:**	**481**	**1380**	**1567**	**1394**	**703**	**1702**

* Starting from 2016, we started to focus on peripheral aneurysm and thrombus as well (PAD/aneurysm/thrombus). ** Since 2017, we have focused only on peripheral aneurysm and thrombus (aneurysm/thrombus). ^#^ AAA tissue is increasingly difficult to obtain due to the fact that open aneurysm repair is more and more frequently replaced by endovascular techniques.

**Table 2:** List of publications resulting from our Munich Vascular Biobank.

Years	AAA	CAROTIS	PAD	∑
**2004–2008**	1	1	1	3
**2009–2013**	17	20	3	40
**2014–2018**	10	23	6	41
**Sum:**	**29**	**44**	**11**	**84**
